# CdS Deposited In Situ on g-C_3_N_4_ via a Modified Chemical Bath Deposition Method to Improve Photocatalytic Hydrogen Production

**DOI:** 10.3390/molecules28237846

**Published:** 2023-11-29

**Authors:** Ligang Ma, Wenjun Jiang, Chao Lin, Le Xu, Tianyu Zhu, Xiaoqian Ai

**Affiliations:** 1 School of Electronic Engineering, Nanjing Xiaozhuang University, Nanjing 211171, China; lgma@njxzc.edu.cn (L.M.);; 2 School of Physics and Information Engineering, Jiangsu Province Engineering Research Center of Basic Education Big Data Application, Jiangsu Second Normal University, Nanjing 210013, China

**Keywords:** monolayer g-C_3_N_4_, CdS, heterojunction, photocatalytic hydrogen production

## Abstract

Ultra-thin two-dimensional materials are attracting widespread interest due to their excellent properties, and they are becoming ideal candidates for a variety of energy and environmental photocatalytic applications. Herein, CdS nanorods are successfully grown in situ between a monolayer of g-C_3_N_4_ using a chemical water bath method. Continuous ultrasound is introduced during the preparation process, which effectively prevents the accumulation of a g-C_3_N_4_ layer. The g-C_3_N_4_@CdS nanocomposite exhibits significantly enhanced photocatalytic activity for hydrogen production under visible-light irradiation, which is attributed to a well-matched band structure and an intimate van der Waals heterojunction interface. The mechanism of photocatalytic hydrogen production is discussed in detail. Moreover, our work can serve as a basis for the construction of other highly catalytically active two-dimensional heterostructures.

## 1. Introduction

With the rapid development of global industry, various problems, for example, climate warming, energy crisis, and environmental pollution, come along [1]. Using catalysts to harness inexhaustible solar energy is a green, sustainable, and promising method [2,3,4,5]. Under the irradiation of sunlight, a photocatalyst is excited to produce electron–hole pairs, and the electrons and holes undergo the reduction reaction and oxidation reaction, respectively, which can decompose organic pollutants into hydrocarbons [6,7,8], split water to produce hydrogen and oxygen [9], and reduce CO_2_ to fuels such as CO and CH_4_ [10]. In addition, excited electrons reduce nitrogen gas to ammonia, which is an important chemical feedstock and is widely used in agricultural waste [11]. In the photocatalytic reaction, the most critical choice is the photocatalyst, which determines the concentration of the electron–hole pairs produced under light irradiation.

In recent years, many semiconductor materials and nanocomposites have been developed in the field of photocatalysis, for example, CdS [12], ZnIn_2_S_4_ [13], PdS [14], and TiO_2_ [15], among others [16]. Among the many semiconductors, graphite-like carbon nitride (g-C_3_N_4_) has become a research hotspot because of its suitable band structure and unique electric, optical, structural, and photochemical properties [17,18,19,20]. It is a non-metallic inorganic n-type semiconductor polymer composed of C and N elements, in which both C and N atoms are hybridized in the form of *sp*^2^ and are connected to form a ring by the *σ* covalent bond, and, between the rings, they are connected by amino groups to form a *π* electron conjugated structure. Therefore, these unique structures of g-C_3_N_4_ can be applied to many fields. In 2009, Wang and his colleagues [21] first discovered that g-C_3_N_4_ produces hydrogen and oxygen under light irradiation, and this application for hydrogen evolution has attracted wide attention. Since then, a wide variety of g-C_3_N_4_-based photocatalysts have been designed to drive various reduction and oxidation reactions under light irradiation [17,22,23,24,25]. Here, the CdS materials have a suitable bandgap width of 2.4 eV and a good visible-light response, but there is serious photo-corrosion, which leads to a severe recombination of photogenerated carriers [6]. If CdS and g-C_3_N_4_ combine to form a heterojunction, there will inevitably be a photocatalytic performance of 1 plus 1 is greater than 2. Therefore, nanocomposites of g-C_3_N_4_ and CdS have been widely reported and studied [22,26,27]. The combination of excellent single-layer g-C_3_N_4_ and CdS will form a fast electron transport channel, and the separation efficiency of photogenerated electrons and holes can be greatly improved, thus showing an excellent rate of photohydrogen production. Jianjun Liu [28] systematically calculated the energy band structure and charge transfer of the heterojunction between g-C_3_N_4_ and CdS using the hybrid density functional approach. They suggested that the contact between CdS and monolayer g-C_3_N_4_ forms a van der Waals heterojunction, which will have an internal electric field that facilitates the separation of the electron–hole pair at the interface. Researchers [18,29] obtained a monolayer of g-C_3_N_4_ using ultrasound, and then they grew CdS using solvothermal, hydrothermal, and other methods. Although monolayer g-C_3_N_4_ obtained via ultrasound is easy to agglomerate to form bulk g-C_3_N_4_ in the process of the hydrothermal growth of other semiconductors, thus improving photocatalytic performance, there are still some limitations. 

In this paper, CdS were grown in situ on monolayer g-C_3_N_4_, which is equivalent to a substrate. In order to better obtain monolayer g-C_3_N_4_, ultrasound was consistently maintained during the chemical bath deposited method, which is a method that has simple equipment and a low cost and allows for easy large-area preparation. Consequently, intimate contact interfaces between g-C_3_N_4_ and CdS were also obtained, which could accelerate the separation of photogenerated carriers. The experimental results show that the photocatalytic performance of the composites improved. The crystal structure, microstructure, and morphology of the composite were analyzed in detail. Moreover, photocatalytic mechanisms were also proposed, and they were demonstrated using characterization methods.

## 2. Results and Discussion

**Figure 1:**
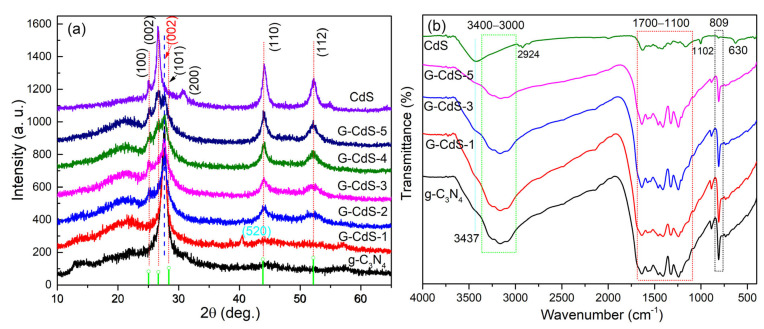
(**a**) XRD patterns and (**b**) FTIR patterns of CdS, g-C_3_N_4_, and G-CdS nanocomposites.

The crystal structures of the CdS, g-C_3_N_4_, and G-CdS nanocomposites were characterized using an X-ray diffractometer (XRD), as shown in Figure 1a. For pure g-C_3_N_4_, two diffraction peaks were found at 12.9° and 27.6°, which can be indexed as (100) and (002) diffraction planes for graphitic materials (JCPDS 87-1526) [21]. The (100) diffraction peaks and (002) diffraction peaks are associated with the in-plane repeated units and periodic graphitic stacking of the conjugated aromatic system [30,31], respectively, which indicates the existence of graphite-like layer structures. The obtained composite photocatalyst exhibited gradually appearing diffraction peaks at 25.1, 26.6, 44.1, and 52.1°, while the intensity of the diffraction peaks related to g-C_3_N_4_ gradually weakened with increasing concentrations of Cd and S. A comparison with the standard PDF card (65-3414) showed that these diffraction peaks are (100), (002), (110), and (112) of the CdS hexagonal wurtzite structure, respectively. The results of XRD indicate that the CdS of the hexagonal wurtzite structure was grown in situ on the g-C_3_N_4_ nanosheets through the CBD process, and the concentration of CdS gradually increased with the increase in Cd and S sources. The characteristic functional groups of the photocatalysts were analyzed using Fourier Transform Infrared (FTIR) spectroscopy. For g-C_3_N_4_, the spectrum reveals several notable features. The prominent absorption peak at 807 cm^−1^ can be attributed to the stretching vibration of heptazine ring units [32], as depicted in Figure 1b. Additionally, the broad band observed in the range of 1100–1700 cm^−1^ corresponds to the stretching vibration mode of the aromatic C-N heterocyclic skeleton, which is characteristic of the typical structure of g-C_3_N_4_. The spectrum for g-C_3_N_4_ also shows peaks in the range of 3000–3400 cm^−1^, indicating the N-H bond stretching vibration of -NH_x_ [33], while the weak peak observed at 3437 cm^−1^ was attributed to the O-H stretching vibration, likely due to the presence of hydroxyl groups or the physical adsorption of H_2_O molecules [34]. As for CdS, the spectrum exhibits distinct peaks at 3437 cm^−1^ and 1622 cm^−1^, corresponding to the surface-adsorbed water molecules [35]. Additionally, the peaks at 1333 cm^−1^ and 1167 cm^−1^ are associated with the stretching vibration peak of Cd-S bonds. The peaks at 2924 cm^−1^ are attributable to the bending vibration of -CH_2_ and -CH_3_ groups [36,37]. Furthermore, in the G-CdS nanocomposites, both partial CdS vibration peaks and partial g-C_3_N_4_ vibration peaks were observed, indicating the successful combination of the two materials without impurities.

**Figure 2:**
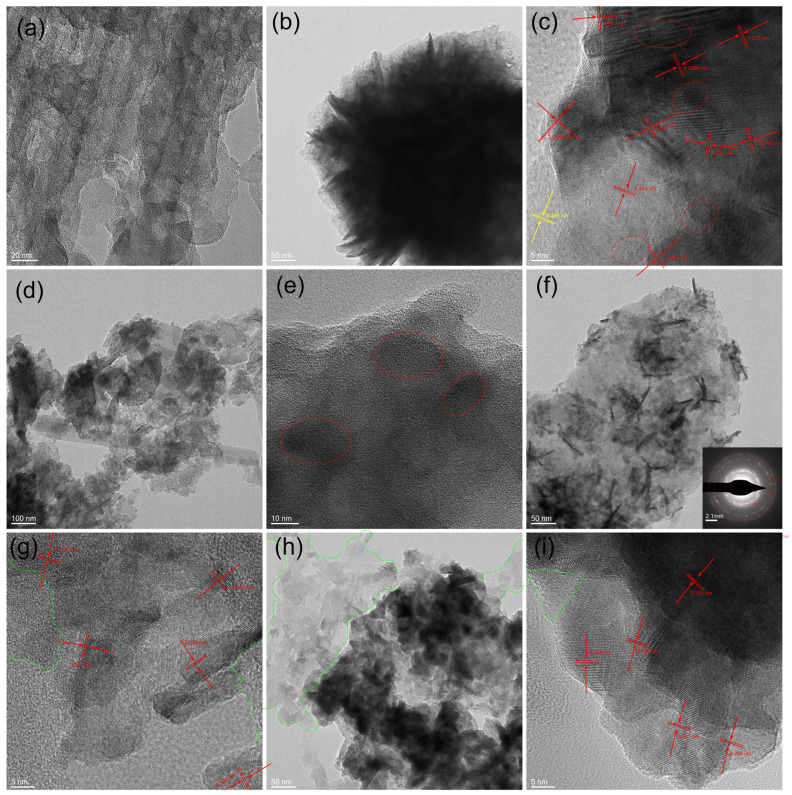
TEM images of (**a**) g-C_3_N_4_ without ultrasound, (**b**) CdS, (**d**) G-CdS-1, (**f**) G-CdS-3, and (**h**) G-CdS-5. HRTEM images (**c**) CdS, (**e**) G-CdS-1, (**g**) G-CdS-3, and (**i**) G-CdS-5. The red circled areas and green circled areas correspond to CdS and g-C_3_N_4_.

The microstructures and morphologies of the samples were characterized and analyzed using transmission electron microscopy (TEM), as shown in Figure 2. Both the amorphous and crystalline g-C_3_N_4_ exhibited an exceptionally thin nanosheet morphology. In contrast, the TEM morphology of pure CdS showed significant agglomeration (Figure 2b). Moreover, in the high-resolution TEM (HRTEM) images of the edge nanoparticles, lattice fringes with spacings of 0.351, 0.337, 0.2065, and 0.292 nm could be observed. The lattice fringes at 0.351 nm, 0.337 nm, and 0.2065 nm correspond to the hexagonal wurtzite structure of CdS, while the lattice fringe at 0.292 nm corresponds to the cubic structure of CdS. This indicates that CdS, in the absence of g-C_3_N_4_, consists of both hexagonal and cubic structures, which is consistent with the XRD results. For the G-CdS nanocomposites, many amorphous quantum dots with sizes around 8 nm emerged on the g-C_3_N_4_ nanosheet structures. This observation is consistent with the amorphous CdS results obtained from the XRD pattern. As the concentration of Cd and S further increased, nanorod-shaped nanowire structures with a length of approximately 50 nm and a width of around 4 nm emerged on the g-C_3_N_4_ nanosheet layer. Further confirmation through HRTEM revealed that these needle-shaped nanowire structures were composed of CdS material with a hexagonal wurtzite structure (the lattice fringes are marked in red in Figure 2). A clear and tight contact interface between g-C_3_N_4_ and CdS was achieved, which implies an intimate heterojunction between the two components, as indicated by the green dashed box in Figure 2g. Compared with Figure 2a, it can be seen that the thickness of the g-CN nanosheets is significantly thinner. This facilitated the effective transfer of charge carriers between the two semiconductors. This indicates that the CdS nanowire structures were grown in situ on g-C_3_N_4_ rather than being a mere physical mixture of the two materials. The high-resolution lattice stripe on the CdS nanorods was 0.333 nm, which means that the nanorods were preferentially grown in the (002) direction. For the G-CdS-5 nanocomposites, the nanorod-shaped CdS nanowire structures further grew in both length and width (Figure 2h). Therefore, according to the TEM morphology, it could be concluded that the concentrations of Cd and S sources influence the morphological evolution of CdS.

**Figure 3:**
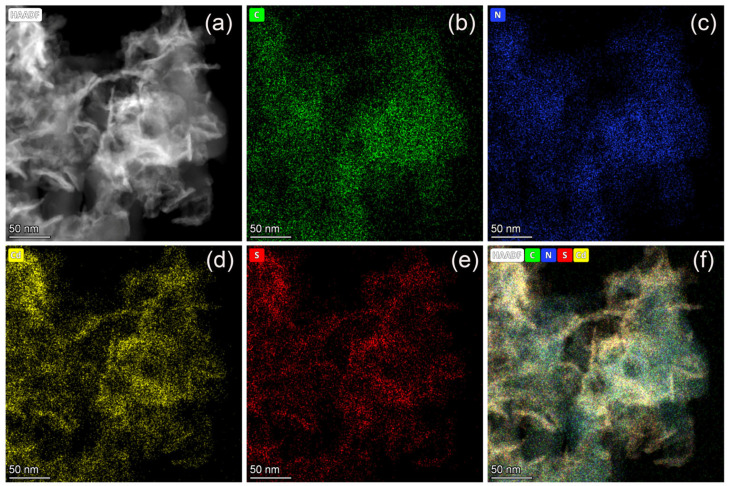
Element mapping of G-CdS-3 nanocomposites: (**a**) high-angle annular dark-field image, (**b**) C element, (**c**) N element, (**d**) Cd element, (**e**) S element, and (**f**) overlay diagram of element mapping.

Moreover, elemental mapping techniques were utilized to investigate the elemental compositions of the G-CdS-3 nanocomposites. As depicted in Figure 3, it was observed that the G-CdS nanocomposites contained C, N, Cd, and S elements with no other impurities detected, which further confirms the successful synthesis of the composite samples. The results also show a uniform spatial distribution of C, N, Cd, and S elements, indicating the homogeneous distribution of the CdS nanowire structures on the surface of g-C_3_N_4_ or between the layers of g-C_3_N_4_.

**Figure 4:**
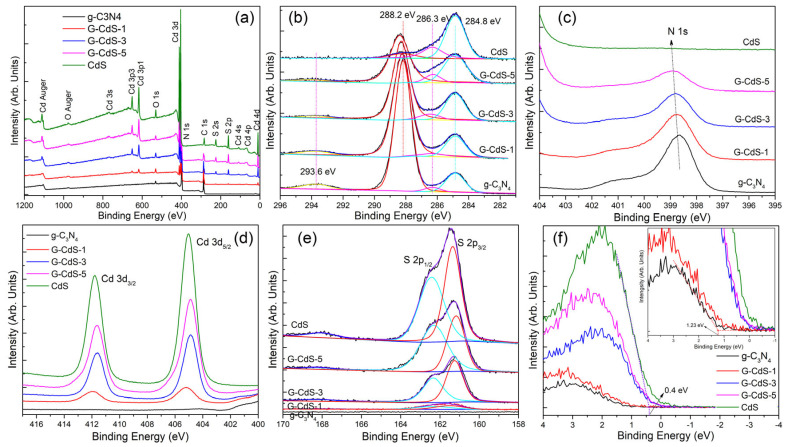
XPS spectra of g-C_3_N_4_, CdS, and G-CdS nanocomposites: (**a**) survey spectrum, (**b**) C 1s, (**c**) N 1s, (**d**) Cd 3d, (**e**) S 2p, and (**f**) valence-band spectra. (**b**,**e**) are the results of Gaussian fitting.

The element compositions and chemical states in the samples were further investigated through X-ray photoelectron spectra (XPS). As shown in Figure 4a, in comparison to the CdS and g-C_3_N_4_ samples, the G-CdS nanocomposites contained Cd, S, C, N, and O elements, and the presence of a slight amount of O element may be attributed to the absorbed oxygen (such as H_2_O and CO_2_) on the surface of the sample, in good agreement with the elemental mapping results. All the high-resolution spectra were calibrated by setting the binding energy of the C-C peak to 284.8 eV. Figure 4b shows the C1s spectrum, which was fitted using Gaussian functions to analyze the types and quantities of functional groups present in the sample. In g-C_3_N_4_, the C1s core-level spectra consisted of four peaks located at 284.8, 286.3, 288.2, and 293.6 eV, which could be attributed to C=C, N≡C- groups [38], N-C=N in typical s-triazine rings [39], and the carbon attached to uncondensed -NH_2_ groups [40], respectively. The strength of the N-C=N bond gradually weakened, which was attributed to the increasing concentration of CdS. At the same time, the position of the bond energy also shifted, indicating that the coupling between g-C_3_N_4_ and CdS became stronger. Figure 4e shows that the N 1s spectrum has three peaks at 398.6 and 400.8 eV, which were attributed to the bi-coordinated N (C=N-C) and N-H bonds, respectively. These peaks also gradually weakened and shifted. With the increase in the CdS concentration, two distinct peaks with binding energies of 404.9 and 411.7 eV appeared, corresponding to the Cd 3d_5/2_ and Cd 3d_3/2_ states, respectively, of the Cd atoms in the Cd-S bonds [6,41,42]. At the same time, the S 2p peak gradually emerged, which could be deconvoluted into two doublets using Gaussian fitting, as displayed in Figure 4e. These two peaks were located at 161.3 (S 2p _3/2_) and 162.3 (S 2p_1/2_), which are characteristic of S species from CdS. It could clearly be seen that the two peaks moved towards a higher binding energy with the increase in the CdS concentration, which means that the crystallization quality and the coupling of the heterojunction further improved. These pieces of evidence, combined with the results of XRD, the FTIR spectra, and TEM, prove that there was a significant heterojunction between CdS and g-C_3_N_4_ in the G-CdS nanocomposites. This indicates that the G-CdS nanocomposite will exhibit excellent performance in terms of charge carrier transport. To determine the band structure, the valence band (VB) spectrum of XPS was measured to obtain the balance band potential (E_vb, XPS_) using VB-XPS plots, as shown in Figure 4f. The intersection of the epitaxial linear part with the *x*-axis provides the E_VB, XPS_ of CdS, and g-C_3_N_4_ with values of 0.4 and 1.23 eV, respectively. Then, the E_VB_ of the corresponding standard hydrogen electrode (E_VB, NHE_) could be calculated as follows [43]: E_VB, NHE_ = *ϕ* + E_VB, XPS_-4.44, where *ϕ* is the work function of the instrument (4.258 eV). Therefore, the E_VB, NHE_ of CdS and g-C_3_N_4_ were calculated as 1.05 and 0.22 eV, respectively. 

**Figure 5:**
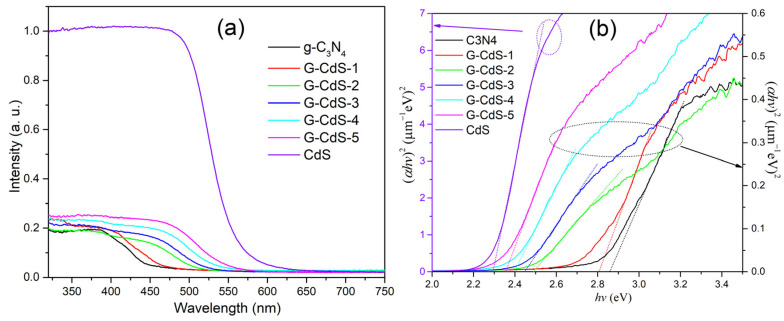
(**a**) UV-vis diffuse reflection spectra, and (**b**) the corresponding Tauc plots of (*αhv*)^2^ versus *hv* for the g-C_3_N_4_, CdS, and G-CdS nanocomposites.

The optical bandgap of a material determines the range of solar light absorption. Therefore, the optical properties were investigated using UV-vis diffuse reflectance spectra (Figure 5a). g-C_3_N_4_ and CdS exhibited sharp absorption band edges at 456 nm and 560 nm, respectively. Compared to g-C_3_N_4_, G-CdS exhibited a slight red shift in its diffuse reflectance spectrum, and the samples appeared darker in color, suggesting the formation of a composite structure of g-C_3_N_4_ and CdS in G-CdS. The redshift observed in the absorption spectra indicates a modification in the electronic band structure of g-C_3_N_4_, resulting from the formation of cyano group defects [38]. Consequently, the absorption edges of the G-CdS samples extend into the visible light region, enhancing the light absorption capability. According to the Tauc formula, the optical bandgap of the samples was calculated. The Tauc formula is as follows [6]: *αhv* = A(*hv* − *E_g_*)^n^, where *E_g_*, *α*, *h*, *v*, A, and n represent the optical bandgap, absorption coefficient, Planck’s constant, incident light frequency, a constant, and n = 1/2 for CdS, respectively. The fitting results, as shown in Figure 5b, indicate that the intersection between the linear extrapolation and the x-axis represents the optical bandgap. The optical bandgaps of g-C_3_N_4_, G-CdS-1, G-CdS-2, G-CdS-3, G-CdS-4, G-CdS-5, and CdS were 2.86, 2.8, 2.49, 2.45, 2.4, 2.36, and 2.29 eV, respectively.

**Figure 6:**
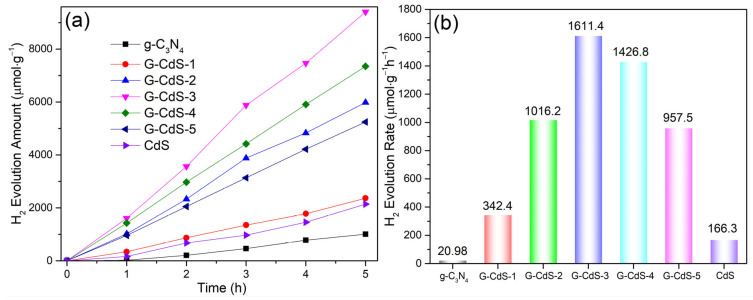
(**a**,**b**) Visible-light photocatalytic H_2_ production rate of g-C_3_N_4_, CdS, and G-CdS nanocomposites.

**Figure 7:**
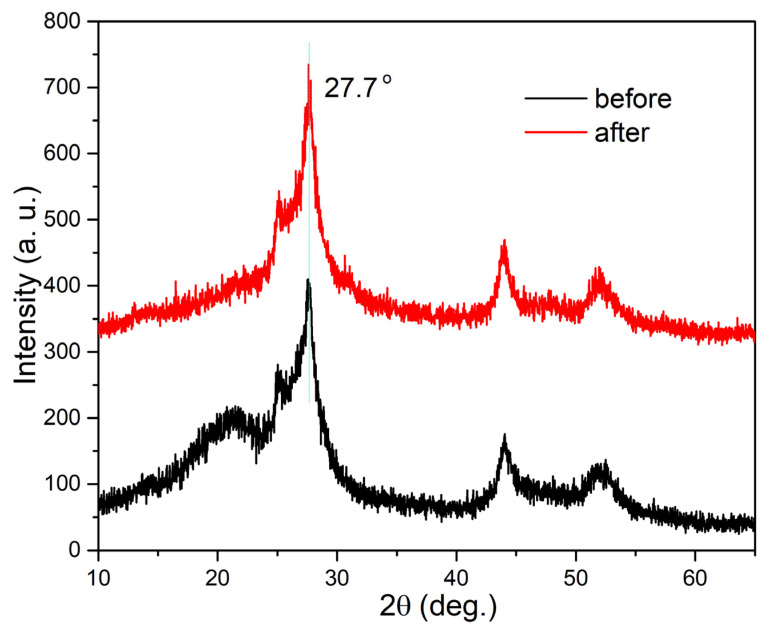
XRD patterns of G-CdS-3 before and after hydrogen production experiments.

Figure 6 shows the photocatalytic hydrogen generation rate of the g-C_3_N_4_, CdS, and G-CdS nanocomposites. It can be seen from the figure that the hydrogen production of all the samples was linear with respect to the time of light irradiation. The g-C_3_N_4_ sample exhibited the lowest hydrogen production performance (20.98 μmol·g^−1^·h^−1^). Moreover, the hydrogen production performance gradually increased with the increase in CdS loading, and the hydrogen production performance of G-CdS-3 reached the maximum (1611.4 μmol·g^−1^·h^−1^), while the performance gradually decayed with the further increase in CdS loading. The hydrogen production of the G-CdS-3 was 76 times higher than that of g-C_3_N_4_ and 10 times higher than that of CdS. It is well known that the photogenerated electrons and holes produced by CdS under light irradiation are inherently strong recombination phenomena. Therefore, when CdS was grown in situ in the interlayer of g-C_3_N_4_, the aggregation of g-C_3_N_4_ decreased, and the specific surface area could be increased at an appropriate concentration, exposing more active sites, effectively increasing the photogenerated carrier separation rate and improving the photocatalytic performance of the nanocomposite. The photocatalytic hydrogen production properties of related g-C_3_N_4_@CdS nanocomposites were investigated, and a comparison of these properties is shown in Table 1. As shown in Table 1, the G-CdS nanocatalysts in this paper demonstrated excellent performance. Furthermore, by measuring the XRD patterns of the G-CdS-3 nanocomposites after the hydrogen production experiment, it was found that the diffraction patterns were basically unchanged compared with those of the fresh sample, as shown in Figure 7. 

By adding a certain amount of cadmium acetate and ammonia water into the ultrasonic g-C_3_N_4_ nanosheet aqueous solution, a cadmium complex, [Cd(NH_3_)_4_]^2+^, was formed in the alkaline environment. Under the action of ultrasound, the [Cd(NH_3_)_4_]^2+^ was uniformly attached to the g-C_3_N_4_ nanosheet. With the addition of thiourea, S^2-^ formed in the alkaline solution, where ammonium acetate acted as a buffer to control the release rate of S. In this way, Cd and S heteronucleated in the layer of g-C_3_N_4_ to form CdS. When the accumulation of Cd and S ions exceeded the solubility of CdS, CdS nanoparticles were deposited on g-C_3_N_4_. In the XRD pattern (Figure 1), the (002) diffraction peak of g-C_3_N_4_ could be significantly shifted to the lower diffraction angle, which means that the layer spacing of g-C_3_N_4_ becomes larger. With the progress of the reaction, CdS grew along the (002) crystal planes, and CdS nanorods were formed. The chemical equation of the reaction is as follows:

Cd + NH_3_ → [Cd(NH_3_)_4_]^2+^

SC(NH_2_)_2_ + 3OH^−^ → 2NH_3_ + CO_3_^2−^ + HS^−^

HS^−^ + OH^−^ → S^2−^ + H_2_O

Cd^2+^ + S^2−^ → CdS

**Scheme 1:**
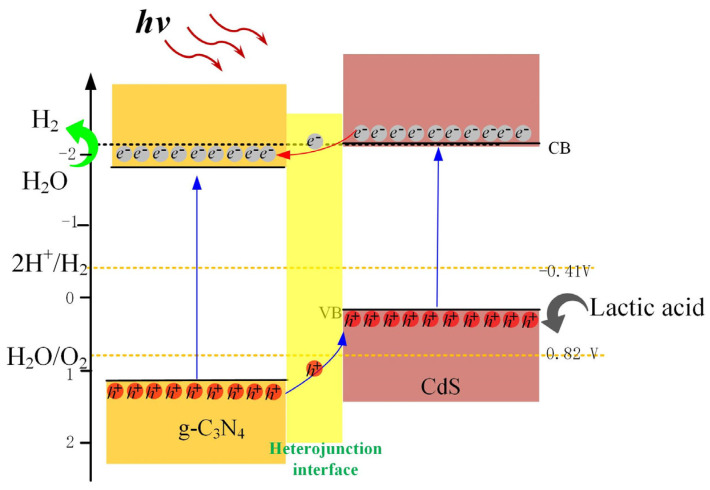
Diagram of the band edge positions G-CdS nanocomposites.

Based on the results of XPS and the UV-vis absorption spectrum analysis, the band structures of the G-CdS nanocomposites are illustrated in Scheme 1. It can be seen that a Type Ⅱ heterojunction formed between CdS and g-C_3_N_4_, which is consistent with the band structure calculated theoretically in the literature [28]. Under light irradiation, electron hole pairs were generated, and the well-matched Type Ⅱ g-C_3_N_4_/CdS heterojunction could realize the positive synergistic effect of accelerating carrier separation and inhibiting CdS corrosion. In addition, during the preparation process, accompanied by ultrasound, g-C_3_N_4_ presented a monolayer or several layer structures, which had a van der Waals heterojunction with CdS. Therefore, the presence of an internal electric field in the heterojunction further promoted the separation of electron–hole pairs at the g-C_3_N_4_/CdS interface [28]. The separation of charge carriers in the G-CdS heterojunctions was also confirmed by photoluminescence and a photochemical test.

**Figure 8:**
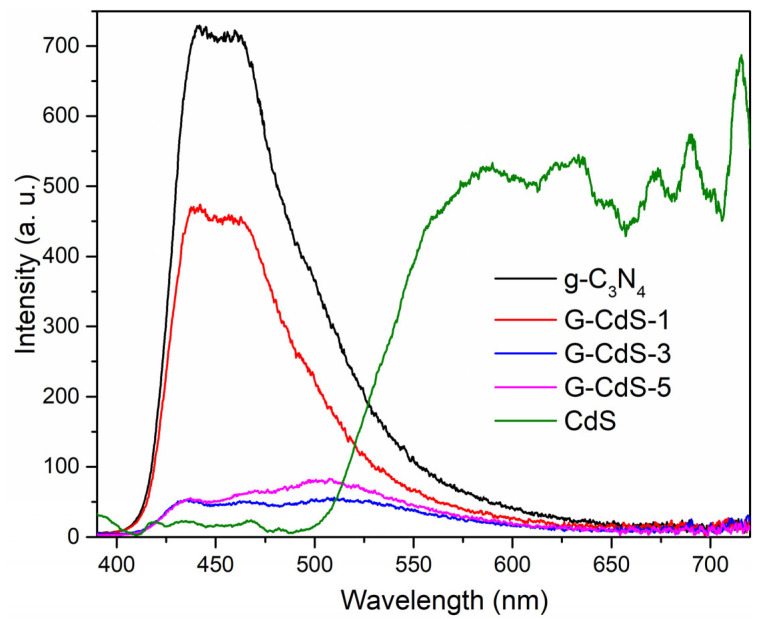
Photoluminescence emission spectra of CdS, g-C_3_N_4_, and G-CdS.

In order to further investigate the luminescent properties of the composite related to the recombination of photogenerated charge carriers under light irradiation, the photoluminescence spectra were measured with an excitation wavelength of 370 nm, as shown in Figure 8. It can be observed in the figure that g-C_3_N_4_ and CdS exhibited strong photoluminescence peaks, while the intensity of the photoluminescence peak in the G-CdS-3 nanocomposites was the weakest. This suggests that the G-CdS-3 sample has a lower probability of photogenerated carrier recombination under light irradiation, indicating higher photocatalytic efficiency.

**Figure 9:**
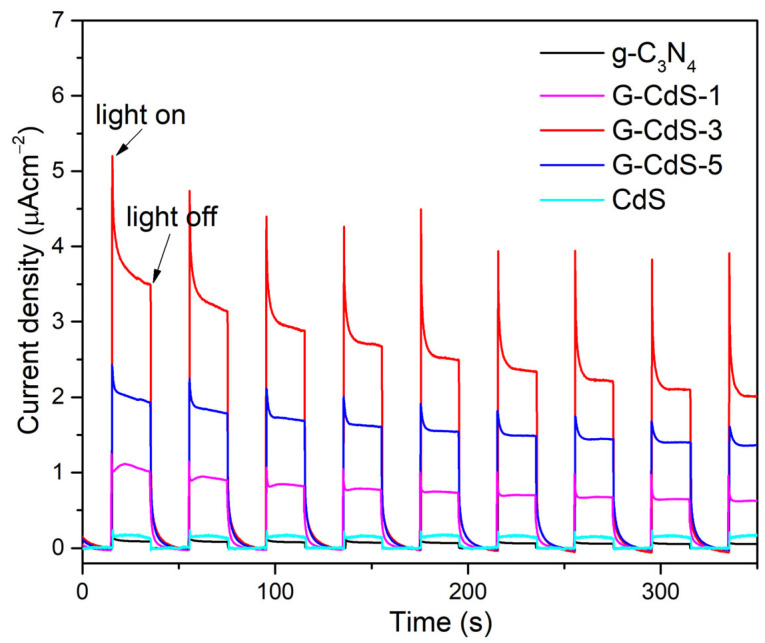
Photocurrent response curves of g-C_3_N_4_, CdS, and G-CdS nanocomposites.

**Figure 10:**
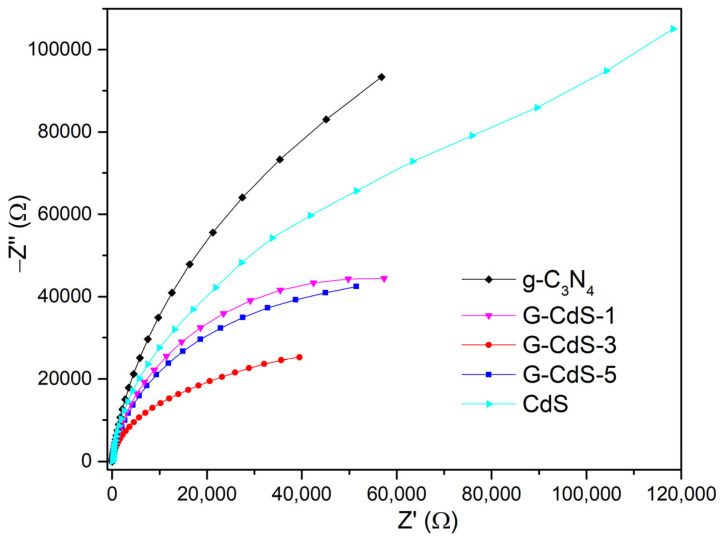
ESI Nyquist plots of g-CN, G-CdS, and CdS.

In addition, the interfacial charge transfer and separation capabilities of the g-C_3_N_4_, CdS, and G-CdS nanocomposites were also investigated in photoelectric chemistry experiments. As shown in Figure 9, the photoelectric response of the G-CdS nanocomposites was higher than that of g-C_3_N_4_ and CdS. Moreover, G-CdS-3 exhibited the best photocurrent density, which had high hydrogen production performance. Furthermore, electrochemical impedance spectroscopy (EIS) was employed to delineate the carrier transport and separation processes, as illustrated in Figure 10. Within EIS spectroscopy, the arc radius serves as a gauge for electron transfer capacity and efficiency in separating photogenerated carriers, and it expedites interfacial charge transfer [48]. Our experimental findings reveal the G-CdS-3 sample exhibits the smallest arc radius, underscoring its superior charge transfer and photogenerated electron–hole pair separation capabilities. This underscores an accelerated interface charge transfer rate within the sample.

## 3. Experimental Section

### 3.1. Materials

Melamine (99%), cadmium acetate dihydrate (Cd(CH_3_COO)·H_2_O), 99.99%), ammonium acetate (CH_3_COONH_4_, 99%), ammonia solution (NH_3_·H_2_O, AR, 25–28%), and Thiourea (CH_4_N_2_S, 99%) were purchased from Aladdin Reagent Co., Ltd. (Shanghai, China), and they were used directly without further purification. Deionized water with 18 MΩ cm was used in our experiment. 

### 3.2. Synthesis of g-C_3_N_4_ Nanosheets

Firstly, 5g melamine powder was heated to 550 °C in an alumina crucible with a lid using a tube furnace at a heating rate of 5 °C/min and kept in the air for 2 h. The collected yellow bulk g-C_3_N_4_ was ground into a fine powder in an agate mortar. Secondly, g-C_3_N_4_ nanosheets were obtained via the thermal etching of bulk g-C_3_N_4_ at 500 °C in the air for 2 h. Finally, the g-C_3_N_4_ nanosheets were washed in deionized water and ethanol three to four times in sequence.

### 3.3. Synthesis of G-CdS Heterojunction

**Scheme 2:**
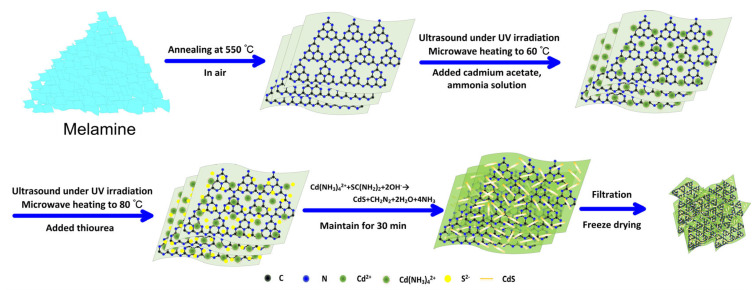
Schematic diagram of the formation process of G-CdS heterostructure.

A schematic diagram of the deposition process of CdS on g-C_3_N_4_ is shown in Scheme 2. The g-C_3_N_4_@CdS (G-CdS) heterojunction was prepared using a modified chemical bath deposition (CBD) method [49] with ultrasonic microwave photocatalytic synthesis. In brief, 500 mg g-C_3_N_4_ and a certain amount of Cd(CH_3_COO)·H_2_O were mixed in 200 mL of deionized water for ultrasonic dispersion. Two hours later, 0.03 M CH_3_COONH_4_ was added, and NH_3_·H_2_O was added to adjust the pH to 11. The abovementioned mixed solution was heated to 60 °C for 30 min. Then, 0.004 M CH_4_N_2_S was added to the solution and heated to 90 °C for 30 min. When the reaction was over, the solution naturally cooled to room temperature. After the reaction, the solution was washed with deionized water and ethanol, and then it was filtered to obtain heterojunction materials. Finally, the obtained nanocomposites were dried at 60 °C for 12 h. The prepared samples were marked as G-CdS-1, G-CdS-2, G-CdS-3, G-CdS-4, and G-CdS-5, indicating the amount of Cd(CH_3_COO)·H_2_O as 0.002 M, 0.004 M, 0.006 M, 0.008 M, and 0.01 M, respectively. As a reference, the synthesis process of CdS is similar to that of G-CdS-3, except that g-C_3_N_4_ is not added.

### 3.4. Characterization

The crystal structures of the g-C_3_N_4_, CdS, and G-CdS nanocomposites were characterized using an XRD with Cu Kα radiation, which operated at a voltage and current of 40 KV and 80 mA, respectively. The morphologies and microstructures of the nanocomposites were measured using TEM, Talos F200X G2, and HRTEM with an accelerated voltage of 200 KV, and super-X model energy dispersive spectroscopy was used to analyze the element distribution. For the TEM test, the ethanol solution containing 1 mg catalyst was dispersed evenly using ultrasound for 10 min, then dropped on the copper net, dried naturally, and measured. The characteristics of the functional groups in the synthetic materials were analyzed using FTIR, Thermo Scientific Nicolet iS20. The changes in the valence state and band structure of the elements in the nanocomposite were measured using XPS on a PHI 5000 Versaprobe Ⅲ spectroscopy instrument with monochromatic Al Kα radiation. Ultraviolet-visible diffuse reflectance spectra were obtained using a Hitachi UH4150 equipped with an integrating sphere. The steady-state photoluminescence was detected using a Hitachi F7000 spectrofluorometer with an excitation wavelength of 385 nm. The photocurrent performance and EIS spectroscopy were examined using a three-electrode electrochemical workstation (CHI660E, ChenHua Instrument Co., LTD, Shanghai, China). The reference electrode was Ag/AgCl, the counter electrode was a Pt plate, and the working electrode was an FTO glass with a catalyst. The electrolyte was a 0.5 M aqueous solution of Na_2_SO_4_.

### 3.5. Evaluation of Photocatalytic H_2_ Production Activity

For the measurement of photocatalytic H_2_ production, a reaction flask was filled with 50 mg photocatalyst, 20% lactic acid aqueous solution (10 mL) as a sacrificial agent, 100 mL of deionized water, and 3% wt Pt as a co-catalyst, using chloroplatinic acid as a Pt source. The mixed solution was ultrasonically dispersed for 30 min to obtain a uniformly dispersed suspension, which was then transferred to a quartz reactor connected to an online trace gas analysis system (Labsolar-6A, Perfectlight, Beijing, China). The system and the reactor were evacuated several times to ensure that the air was completely removed. The reactor was irradiated using a 300 W Xe arc lamp source, and the wavelength of the incident light was regulated by using a 420 nm long pass cut-off filter. The temperature of the reaction solution was maintained at 5 °C with a constant temperature water cooling system. The concentration of photocatalytic H_2_ production after light irradiation was analyzed using an online gas chromatograph (GC9720PLUS, Fuli instruments, Zhejiang, China) with a thermal conductive detector. After the photocatalysis, the photocatalyst was centrifuged, washed several times, and then vacuum dried at 60 °C.

## 4. Conclusions

In summary, a van der Waals heterojunction was successfully fabricated by introducing a continuous ultrasonic step in the process of the chemical bath deposition method. Continuous ultrasound prevented g-C_3_N_4_ from agglomerating, and it remained in a monolayer or ultra-thin state. The microstructure, morphology, and optical properties of the G-CdS heterojunction were characterized using XRD, SEM, TEM, and UV absorption spectra. The results show that CdS of a hexagonal wurtzite structure grew preferentially on g-C_3_N_4_, showing a nanorod structure. Moreover, there was a clear and tight contact interface between g-C_3_N_4_ and CdS, as well as a tight heterojunction between the two components, which helped to efficiently transfer charge carriers between the two semiconductors. Photocatalytic H_2_ production was also studied, and G-CdS-3 showed excellent photocatalytic performance; the catalytic mechanism was revealed using a Type Ⅱ mechanism. PL and photocurrent spectra proved that a Type Ⅱ heterojunction can accelerate carrier separation, reduce recombination, and improve photocatalytic H_2_ production. 

## Figures and Tables

**Table 1:** Comparison of H_2_ evolution performance between G-CdS-3 and other reported photocatalysts.

Catalyst	Synthesis Method	Dosage (mg)	Type of Light Source	Sacrificial Reagent	H_2_ Evolution Rate (μmol·g^−1^·h^−1^)	Refs.
Pd-CdS/g-C_3_N_4_	hydrothermal method and borohydride reduction method	50	300 W Xe arc Lamp (λ > 400 nm)	0.5 M Na_2_S and 0.5 M Na_2_SO_3_	293.0	[44]
CdS/g-C_3_N_4_	photodeposited method	10	300 W Xe arc Lamp (λ > 420 nm)	0.5 M Na_2_S and 0.5 M Na_2_SO_3_	56.9	[45]
CdS/Au/g-C3N4	photodeposited method	100	Xenon Lamp (λ > 420 nm)	10 mL methanol	19.02	[46]
CdS/g-C3N4	hydrothermal synthesis	10	300 W Xe arc Lamp (λ > 400 nm)	20 mL triethanolamine	216.48	[47]
G-CdS	modified CBD method	50	300 W Xe arc Lamp (λ > 420 nm)	20% lactic acid aqueous solution	1611.4 μmol·g^−1^·h^−1^	This work

## Data Availability

Data are contained within the article.
